# The *Drosophila* Actin Regulator ENABLED Regulates Cell Shape and Orientation during Gonad Morphogenesis

**DOI:** 10.1371/journal.pone.0052649

**Published:** 2012-12-26

**Authors:** Hiroko Sano, Prabhat S. Kunwar, Andrew D. Renault, Vitor Barbosa, Ivan B. N. Clark, Shuji Ishihara, Kaoru Sugimura, Ruth Lehmann

**Affiliations:** 1 HHMI and Developmental Genetics Program, Skirball Institute of Biomolecular Medicine, Department of Cell Biology, New York University Medical Center, New York, New York, United States of America; 2 Center for Systems Biology at Edinburgh, Edinburgh, United Kingdom; 3 Graduate School of Arts and Sciences, The University of Tokyo, Tokyo, Japan; 4 PRESTO JST, Saitama, Japan; 5 Institute for Integrated Cell-Material Sciences, Kyoto University, Kyoto, Japan; 6 RIKEN Brain Science Institute, Saitama, Japan; Stockholm University, Sweden

## Abstract

Organs develop distinctive morphologies to fulfill their unique functions. We used *Drosophila* embryonic gonads as a model to study how two different cell lineages, primordial germ cells (PGCs) and somatic gonadal precursors (SGPs), combine to form one organ. We developed a membrane GFP marker to image SGP behaviors live. These studies show that a combination of SGP cell shape changes and inward movement of anterior and posterior SGPs leads to the compaction of the spherical gonad. This process is disrupted in mutants of the actin regulator, *enabled (ena)*. We show that Ena coordinates these cell shape changes and the inward movement of the SGPs, and Ena affects the intracellular localization of DE-cadherin (DE-cad). Mathematical simulation based on these observations suggests that changes in DE-cad localization can generate the forces needed to compact an elongated structure into a sphere. We propose that Ena regulates force balance in the SGPs by sequestering DE-cad, leading to the morphogenetic movement required for gonad compaction.

## Introduction

During organogenesis, cells undergo concerted morphogenetic events, including migration, rearrangement, adhesion, shape changes, and cell death, to confer a unique shape and function to the forming organ. Each event occurs at a specific time and place during development, and requires extensive communication between cells. The cellular and molecular mechanisms underlying these morphogenetic events have remained largely elusive.

**Figure 1:**
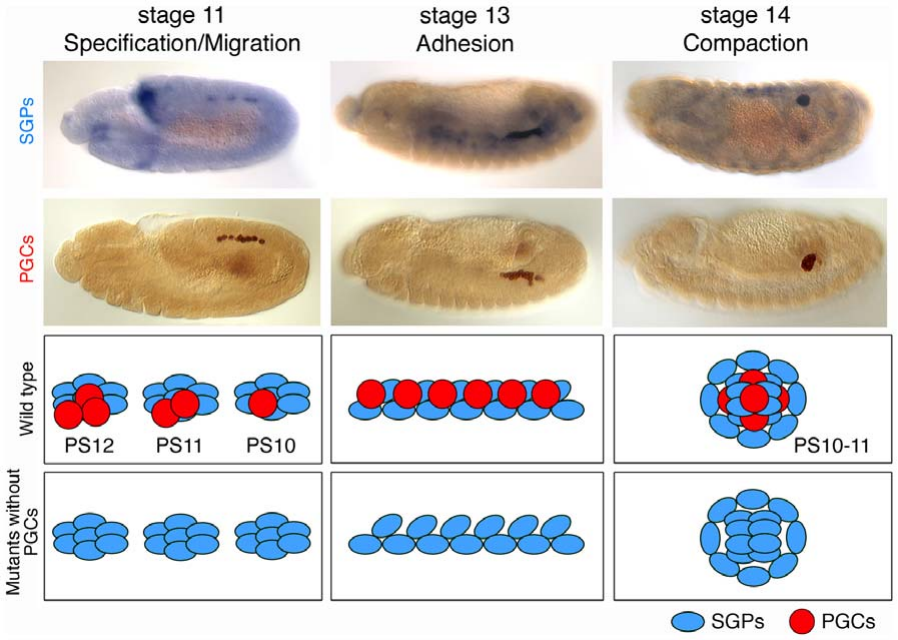
Schematic drawing of gonad formation in wild type and mutants without PGCs. (Top and middle panels) Gonad coalescence in wild type. SGPs (blue) are specified as three clusters in parasegments (PSs) 10–12. At stages 11–12, PGCs (red) migrate to associate with somatic gonads. At stage 13, SGPs and PGCs align in the future gonad region. At stage 14, SGPs and PGCs undergo compaction to form the embryonic gonad. (Bottom panels) Normal gonad coalescence in mutants without PGCs. In the absence of PGCs, SGPs are specified normally and are able to complete gonad coalescence.

We chose *Drosophila* embryonic gonads to study the mechanisms of organ morphogenesis. Gonads are paired organs, comprised of 10–15 primordial germ cells (PGCs) and 60–70 somatic gonadal precursors (SGPs) [1], [2], that exhibit complicated morphogenesis, making *Drosophila* gonad formation an excellent model to study organ formation. PGCs are formed at the posterior pole of the newly formed embryo (reviewed in [3]) and, during gastrulation, are carried into the embryo in close proximity to the posterior midgut primordium. PGCs then pass through the midgut primordium and actively migrate towards the SGPs [4], [5]. Once the PGCs reach the SGPs at stage 11, they cease migration. The SGPs are specified in three separate bilateral clusters in the mesoderm of parasegments (PS) 10, 11, and 12 (Figure 1, left panels) [6], [7], [8]. During stages 11–13, the individual SGP clusters merge to form one elongated gonad primordium (Figure 1, middle panels), which then compacts, during stages 13–14, into the embryonic gonad located in PS10 (Figure 1, right panels).

It has been proposed that gonad coalescence is dictated by the SGPs as it proceeds normally in the absence of PGCs (Figure 1, bottom panels) [6]. Consistent with this observation, previous genetic screens for gonad formation defects identified genes expressed in somatic rather than germline cells [9], [10], [11]. Most of these “somatic genes” encode transcription factors involved in the specification of the lateral mesoderm; the common precursor of both the SGPs and fat body [11], [12]. The GATA-like transcription factor, *serpent (srp)*, promotes fat body development in parasegments (PS) 4–9, while *abdominal A (abd A)* represses *srp* expression in PS10, 11, and 12, allowing SGP formation in the posterior abdominal segments [11], [12].

Unlike SGP specification, SGP morphogenesis is poorly understood. At the onset of gonad coalescence, the PGCs and SGPs align from PS10 to 12 before undergoing compaction to form the spherical gonad in PS10. During these processes, the SGPs extend long cytoplasmic extensions to encapsulate the PGCs [13]. The adhesion protein DE-cadherin (DE-cad) encoded by *shotgun (shg)* is expressed in both PGCs and SGPs [13]. In migrating SGPs, DE-cad is detected at levels similar to those in the surrounding tissues, such as the fat body. By the time the SGPs reach the future gonad region, DE-cad is upregulated compared to surrounding tissues. In *shg* mutants, SGPs are specified and migrate normally, but fail to complete compaction, leaving the gonad extended. The *shg* mutation also disrupts the formation of cytoplasmic protrusions in SGPs, preventing PGC ensheathment [13]. Mutants of a zinc transporter, *fear of intimacy (foi)*, show a similar gonad coalescence phenotype to *shg* mutants [14]. *foi* was shown to be required for the transcription, mRNA stability, and post-transcriptional up-regulation of *DE-cad* in SGPs [13], [15]. *foi* mutant embryos with restored DE-cad expression showed normal gonad coalescence, suggesting that Foi functions mostly through the regulation of *shg* [13]. A recent genetic screen has identified several additional genes, including *slit* and *roundabout* (*robo*), required for gonad formation [16].

In this study, we show that Enabled (Ena) is required for gonad compaction in cooperation with DE-cad. *In vitro* studies have shown that Ena/VASP proteins promote elongation of actin filaments by shielding barbed ends from an actin branching factor, Capping protein [17], [18]. By regulating the geometry of actin filament network, Ena/VASP proteins affect protrusive behavior of lamellipodia and filopodia, thereby regulate cell migration [19], [17]. Loss of Ena in *Drosophila* disturbs axon guidance [20], dorsal closure of epithelial cells [21], [22], and migration of border cells and hemocytes [23], [24]. Ena/VASP proteins are also localized at Cadherin-mediated junctions. In keratinocyte and mammary cells, Ena/VASP proteins are recruited to the cell-cell contacts and regulate actin cytoskeleton [25], [26]. In *Drosophila* follicular epithelium, Ena is enriched at the adherens junction leading to apical actin filament formation and the stabilization of the junction [27]. These observations show that Ena has important roles in the adherens junction; however, how Ena cooperates with Cadherin in morphogenesis has been poorly understood.

To address this question, we investigated the interaction of Ena and DE-cad in gonad morphogenesis. Using live imaging, we show that wild-type SGPs change their shape and move inward in the anterior and posterior regions to give the gonad its spherical appearance. This process is disrupted in *ena* mutants resulting in an elongated gonad. We demonstrate that Ena regulates SGP shape and positioning. Moreover, *ena* affects DE-cad localization within SGPs during gonad compaction. Using the cellular parameters established in our study, we develop a mathematical simulation of gonad coalescence. In this model, changes in SGP-SGP adhesion, probably enhanced by DE-cad *in vivo*, are sufficient to produce forces capable of generating a spherical gonad. Taken together, we propose that Ena contributes to the intracellular localization of DE-cad to confer the adhesive force on SGPs for proper gonad compaction.

## Results

### *ena* is required for gonad coalescence

**Figure 2:**
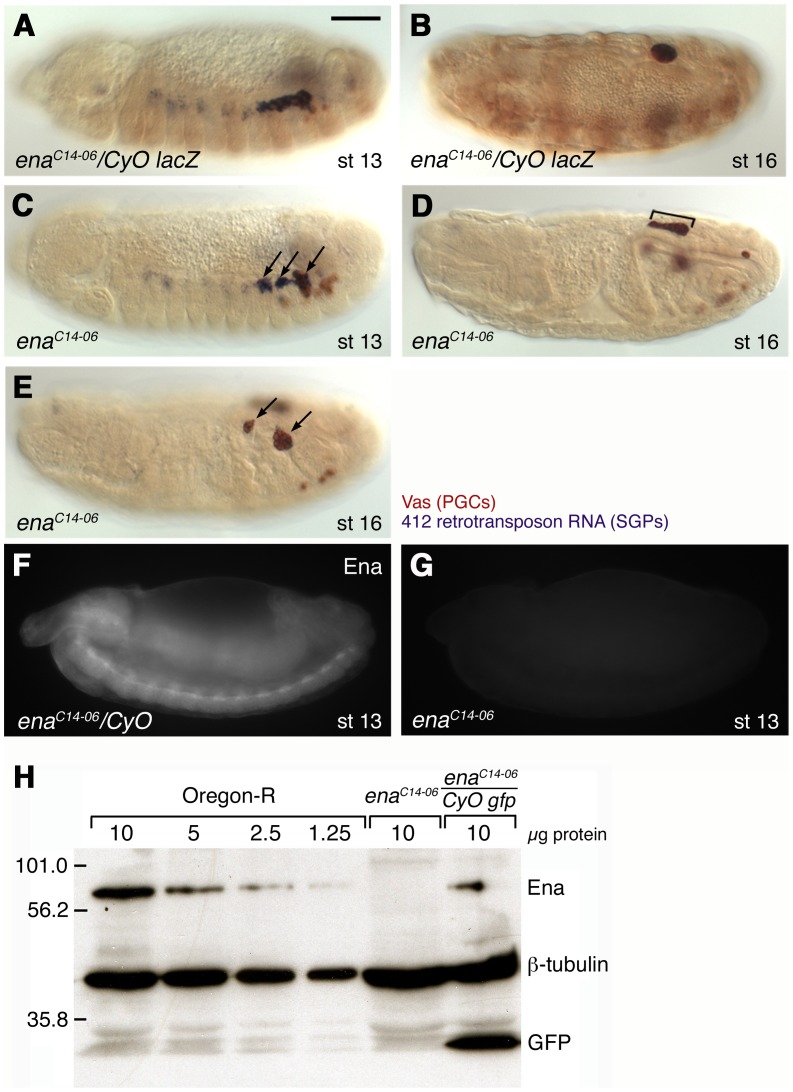
The *ena^C14-06^* allele affects gonad coalescence. (A–E) SGPs were detected by *in situ* hybridization with a 412 retrotransposon RNA probe (blue). PGCs were labeled with anti-Vasa (Vas) antibody (brown). The brown signal in a segmental pattern is β-galastosidase from the balancer chromosome. In wild type, individual SGP clusters align with PGCs at stage 13 (A) and undergo coalescence to form tightly associated embryonic gonads at stage 14 to 16 (B). In *ena^C14-06^* mutants, individual SGP clusters fail or delay to adhere at stage 13, remaining instead in three clusters (arrows in C). Subsequently, mutant gonads fail to complete coalescence resulting in elongated (bracket in D) or split gonads (arrows in E). (F–H) Ena protein detected with anti-Ena antibody. Ena protein is expressed ubiquitously in the wild type (F). Ena protein was not detected in *ena^C14-06^* mutants either by tissue-immuno fluorescence (G) or Western blotting (H). Scale bar in (A) represents 50 µm.

We performed an EMS mutagenesis on the right arm of the second chromosome to identify mutations affecting gonad formation [28], and identified a new allele of *enabled* (*ena*), *ena^C14-06^* (see Materials and Methods). In homozygous *ena^C14-06^* mutant embryos, SGPs were specified normally as three clusters in the mesoderm, and PGCs were associated with all three clusters. However, these clusters failed to adhere to each other at stage 13, when wild-type SGPs aligned into one primordial gonad (Figure 2A, C). At stage 16, wild-type SGPs and PGCs undergo compaction to generate a spherical embryonic gonad, while *ena* mutant gonads remain elongated or split (Figure 2B, D, and E). From stage 13 onward, some mis-migrating PGCs were observed outside the gonad in *ena* mutants (Figure 2C–E).

Antibody staining showed that the Ena expression, observed in wild type control, is abolished in the mutant embryos (Figure 2F and G). Ena protein was undetectable on a Western blot of embryonic protein extracts, suggesting that *ena^C14-06^* is a strong loss-of-function allele of the *ena* gene (Figure 2H).

### *ena* acts in the soma during gonad coalescence

**Figure 3:**
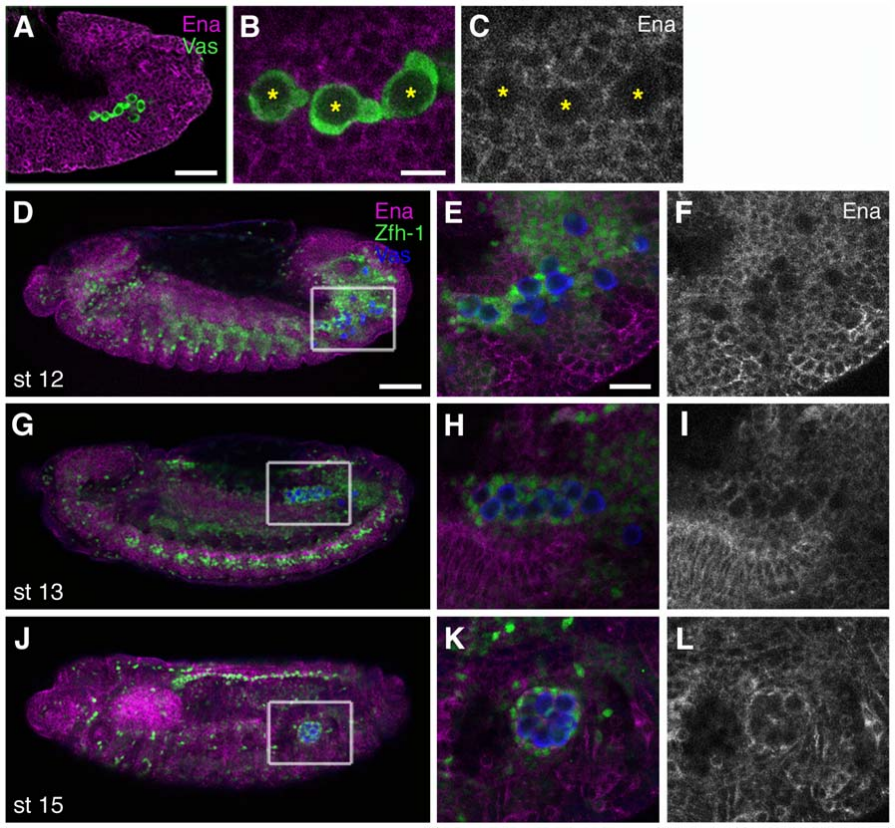
Ena is expressed in the SGPs during gonad coalescence. (A–C) Stage-12 embryo stained with the anti-Ena antibody (magenta in A–B, white in C) and anti-Vas antibody to detect PGCs (green). The posterior half of the embryo is shown in (A), migrating PGCs are magnified in (B, C) (asterisks). Ena protein is found at the cell membranes (A) but not in migrating PGCs (B, C). (D–L) Ena expression during gonad coalescence. Wild-type embryos were stained with anti-Ena (magenta), anti-Zfh-1 antibodies, which mark the nuclei of the fat body and SGPs (green), and anti-Vas (PGCs in blue). Ena levels in the SGPs were indistinguishable from those in the surrounding tissues (D, high magnification in E, F). Ena expression in the SGPs was more pronounced in the mature gonad (G, J, high magnification in H–I and K–L). The scale bars in (A and D), (B), and (E) represent 50 µm, 10 µm, and 20 µm, respectively.

**Figure 4:**
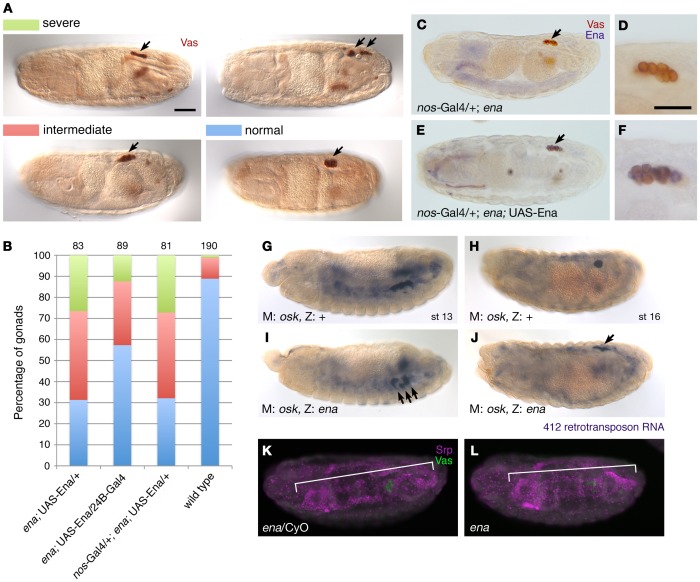
*ena* functions in the soma for proper gonad coalescence. (A–F) Rescue experiment of *ena* mutants. The coalescence phenotype at stages 15–16 was categorized into severe (green), intermediate (red), and normal classes (blue) as illustrated in (A), and number of gonads scored is indicated above the graph (B). Germline expression of Ena by *nanos (nos)*-Gal4 did not show any rescue activity (B) albeit the Ena protein derived from the transgene was detected in PGCs (blue in E and F). Ena expression in the mesoderm by 24B-Gal4 partially rescued the compaction phenotype in the mutants (B). (G–J) *ena* function was tested in a maternal *oskar* mutant background lacking PGCs. The 412 retrotransposon probe was used to label SGPs (blue). In maternal *oskar* mutants, SGPs were specified normally and compacted into spherical ‘gonads’ (G and H). In *ena* (zygotic), *oskar* (maternal) double mutants, SGP clusters failed to adhere at stage 13 (I), resulting in elongated gonads similar to those in zygotic *ena* mutants (J). (K–L) The stage-15 embryos were stained with anti-Serpent and anti-Vas antibodies to detect the fat body (magenta, bracket) and PGCs (green). The fat body appears unaffected by the *ena* mutation. Scale bars in (A) and (D) represent 50 µm.

In order to identify tissues in which *ena* functions, we examined the expression pattern of the Ena protein in wild-type embryos. Ena protein is broadly expressed in the embryo and is enriched at cell borders (Figure 3A). It has been reported that Ena family protein are localized at actin-dependent leading edge structures in migrating hemocytes and in epithelial cells during dorsal closure [24], [21]. Mammalian homologues of Ena are also detected in lamellipodia and filopodia in cultured cells [17], [29], [30]. However, we did not detect Ena at the leading edge of PGCs (Figure 3B and C). We attempted to rescue the gonad compaction phenotype in *ena* mutants by expressing a wild-type *ena* transgene in either the germline or soma. In this assay, we categorized the gonad compaction phenotype into three classes; severe (gonads remain extended or split), intermediate (gonads are not fully compacted/moderately elongated), and normal (Figure 4A). *nanos (nos)*-Gal4-driven *ena* expression in the PGCs did not alter the percentage of gonads in each of the three classes (Figure 4B–F), suggesting that *ena* is not required in PGCs during gonad formation.

In contrast, Ena is clearly expressed in the SGPs. At stage 12, Ena expression levels in SGPs were similar to those in other tissues (Figure 3D–F), before increasing gradually in the SGPs of the coalesced gonad at stage 15 (Figure 3G–L). To test whether Ena is required for gonad coalescence independently of PGCs, we analyzed the *ena* phenotype in the absence of PGCs. To remove PGCs, we used maternal-effect mutations in the *oskar (osk)* gene [31]. In embryos from *osk^CE4^/osk^301^* mutant mothers (hereafter referred as *osk^M-^*), SGPs are specified normally but PGCs are absent (Figure 4G and H) [6]. We generated embryos that were maternally mutant for *osk* and zygotically mutant for *ena* (hereafter referred as *osk^M-^, ena^Z-^*). In *osk^M-^, ena^Z-^* mutants, SGP clusters showed defects or delays in adhesion at stage 13 and failed to complete compaction, remaining extended or split in the later stages, thus resembling the *ena* single mutant phenotype (Figure 4I and J). Ena expression driven by the panmesodermal-GAL4 driver, 24B-GAL4, partially rescued the defects seen in *ena* mutant gonads (Figure 4B). The expression of Ena with the *Dsix4*-GAL4 driver, which activates gene expression in the SGPs, fat body and somatic muscle, also partially restored gonad compaction in the mutants (data not shown). The partial rescue is likely due to failure of the GAL4 driver to correctly recapitulate the levels or expression pattern of the endogenous gene (see also [32], [20]). Taken together, these results demonstrate that *ena* acts in the soma for proper gonad coalescence. The lack of a specific GAL4 driver for SGPs prevented us from testing whether *ena* is required specifically in the SGPs. However, the *ena* mutation apparently does not affect morphogenesis of the fat body, which surrounds the gonad (Figure 4K and L), indicating that the compaction defects are not a secondary consequence of defects in adjacent tissues.

### *ena* is required to generate the inward movement of SGPs necessary to shape the gonad

**Figure 5:**
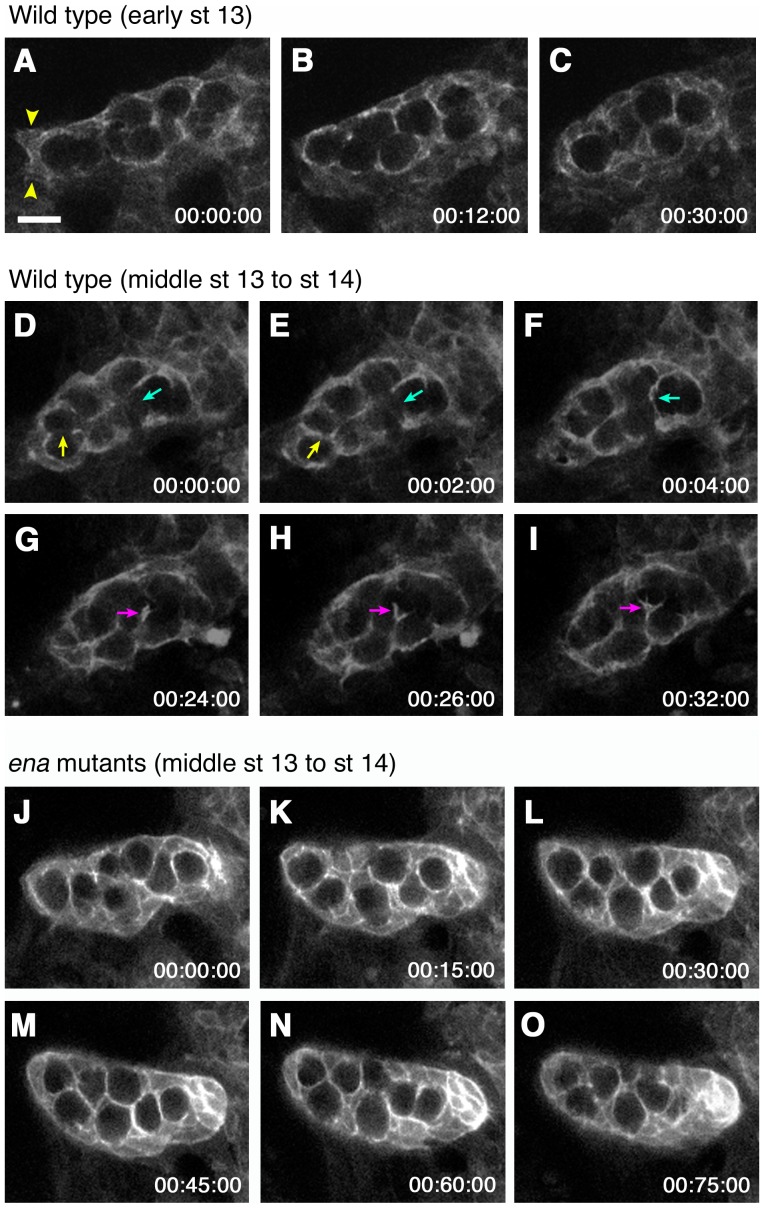
Live imaging of the SGPs in wild type and *ena* mutants. SGPs labeled with P*_D-six4_-egfp::moesin* were imaged in wild-type and *ena* mutant embryos. (A–I) Still images from Movies S1 (A–C) and S2 (D–I) in wild type. At early stage 13, some SGPs had broad migratory protrusions (arrowheads in A), which were lost as gonad coalescence proceeded, and SGPs in the anterior and posterior regions moved to the middle of the gonad (B–C). During coalescence, the SGPs extended protrusions between PGCs (A–I). These protrusions were highly dynamic, and elongated, branched, and connected with each other to encapsulate the PGCs (arrows in D–I). (J–O) Still images from Movie S3 in *ena* mutants. The *ena* mutant gonad imaged in (J–O) displayed moderate phenotype similar to the gonad shown in Figure 6C and D. The mutant SGPs normally ensheathed PGCs; however, the anterior and posterior SGPs were unable to move inward resulting in incomplete coalescence. The scale bar in (A) represents 10 µm.

To analyze gonad coalescence at a cellular level, we generated a reporter construct, P*_D-six4_-egfp::moesin*. In P*_D-six4_-egfp::moesin*, the fusion protein of EGFP and the F-actin-binding C-terminal domain of Moesin is expressed under the control of the *D-six4* promoter, which is located in the third intron of the *D-six4* gene (Figure S1) [33], [34]. *D-six4* expression is detected broadly in the mesoderm at stage 9 and is gradually restricted to the SGPs by the late embryonic stages [35]. P*_D-six4_-egfp::moesin* labels the SGP cell membrane and its expression did not alter wild-type or mutant gonad morphogenesis, enabling us to observe SGP behavior. We first observed the behavior of wild-type SGPs during gonad coalescence using high-resolution live imaging. At early stage 13, we observed broad migratory extensions, possibly required for the initial merger of SGPs from individual clusters (Movie S1, arrowheads in Figure 5A). During gonad coalescence, these protrusions were down-regulated; while the SGPs started extending long, thin protrusions towards the PGCs (Movie S1, Figure 5B and C). These thin protrusions were dynamic, elongating, branching, and connecting with each other to form a meshwork encapsulating the PGCs (Movie S2, Figure 5D–I). Anterior and posterior SGPs moved towards the middle of the gonad to form a spherical shape (Movies S1 and S2, Figure 5A–I). In *ena* mutants, SGPs failed to reposition to the middle, leaving the gonad elongated similar to the immature gonad (Movie S3, Figure 5J–O).

### *ena* is dispensable for the formation of SGP cytoplasmic protrusions

**Figure 6:**
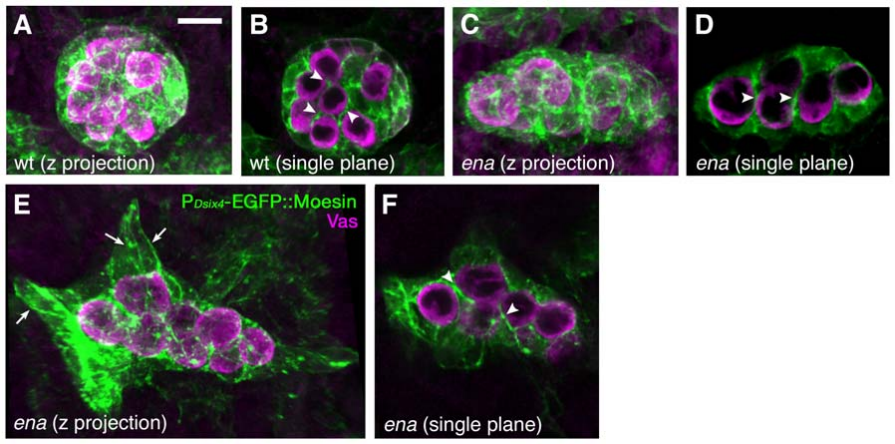
*ena* is dispensable for the formation of cytoplasmic protrusions in SGPs. (A–F) Wild-type and *ena* mutant embryos with the P*_six4_-egfp::moesin* transgene were double stained with anti-GFP (green) and anti-Vas (magenta) antibodies. Labeled gonads at stages 15–16 were scanned and rendered into 3D images. Z projection (A, C, E) and selected single frames (B, D, F) are presented. In the wild-type gonad, SGPs extended numerous protrusions to ensheath PGCs (A, arrowheads in B). In *ena* mutants, SGPs appeared to have normal cytoplasmic extensions surrounding the PGCs (C, E, arrowheads in D, F). Some mutant SGPs, mostly in the anterior region, severely extend (arrows in E), resulting in an irregularly shaped gonad.

To clarify how *ena* regulates gonad compaction, we examined whether *ena* mutations affect the formation of actin-rich-cytoplasmic protrusions in SGPs. We stained embryos harboring the P*_D-six4_-egfp::moesin* transgene with anti-GFP and anti-Vas antibodies and all SGP protrusions in the gonad were visualized in three-dimensional projections. Unexpectedly, the number of SGP protrusions appears to be normal in *ena* mutant gonads (compare Figure 6A, C, and E), extending between the PGCs as in the wild type (compare Figure 6B, D, and F). These results suggest the relatively normal formation of actin filaments in *ena* mutants; however, it does not rule out the possibility that *ena* regulates microstructure of actin filaments as *ena* is specifically involved in the elongation of actin filaments.

### *ena* affects SGP cell rearrangement and changes in cell shape

**Figure 7:**
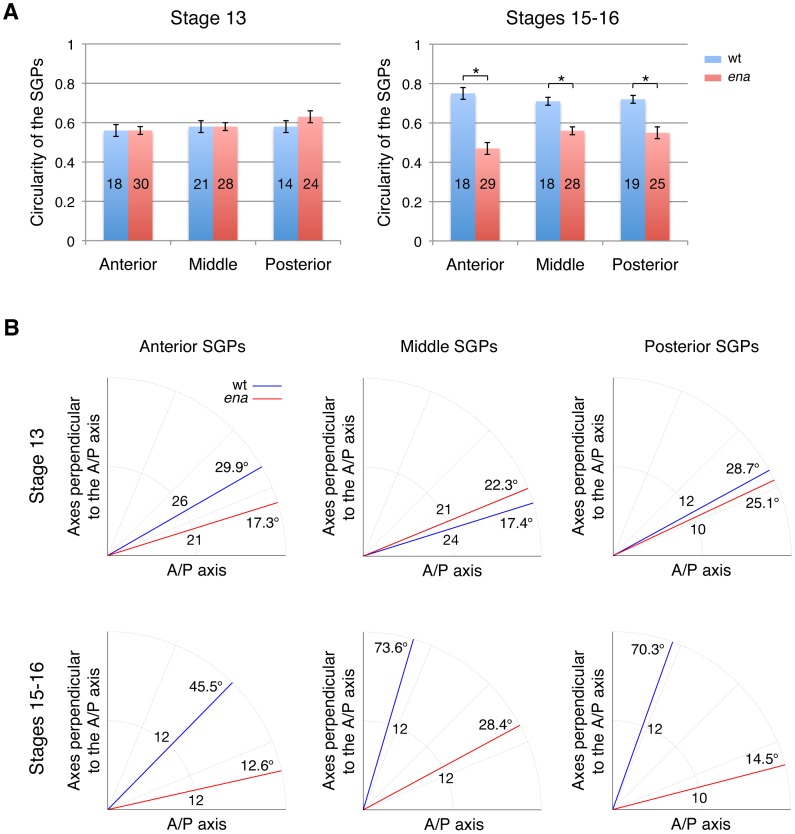
*ena* is required for rearrangement and cell shape changes of SGPs. (A) The circularity of individual SGPs was calculated in the anterior, middle, and posterior regions of the gonad. Circularity (see Materials and methods) was similar between the wild type and mutants at stage 13. However, at stages 15–16, circularity increased with compaction in wild-type SGPs. In *ena* mutants, SGP circularity was unchanged in the later stages, indicating that mutant SGPs remain extended (*P<0.0001). The number of cells examined is shown in the bar. (B) SGP orientation relative to the anterior-posterior (AP) axis of the gonad was measured. At stage 13, wild-type and *ena* mutant SGPs were oriented along the AP axis of the gonad. At stages 15–16, the long axis of wild- type SGPs were oriented away from the AP axis; however, *ena* mutant SGPs remained aligned with the AP axis. The number of cells examined is shown in the middle of the graph. Error bars in (A) represent standard error.

Next, we examined SGP shape and orientation of individual SGPs during gonad compaction at stages 13 and 15–16 in three regions along the gonads' longitudinal axis (Figure 7A and B). To describe changes in SGP cell shape, we calculated their circularity, where a value of 1 indicates perfect circularity. At stage 13, wild-type SGPs attained a polygonal shape with a 0.6 circularity value (Figure 7A, left). As the gonad compacted at stages 15–16, the cells rounded and the value increased to 0.7–0.8 (Figure 7A, right). In contrast, the circularity value of the mutant SGPs remained around 0.6 even in the later stages, indicating that the mutant SGPs remain extended (Figure 7A). In some mutant embryos, anterior SGPs were severely extended (arrows in Figure 6G), which is reflected in the lower circularity of the anterior SGPs in the mutant gonad (P<0.05, Figure 7A, right). We also examined the orientation of individual SGPs within the gonad by comparing the angle of the long axis of an individual SGP relative to the anterior-posterior (AP) axis of the gonad. At stage 13, the long axis of individual SGPs was aligned with the AP axis of the gonad in both wild type and mutants, with an average angle of 21°–25° away from the AP axis of the gonad (Figure 7B, upper graphs). At stages 15–16, the long axis of wild-type SGPs pointed away from the gonad AP axis forming a 46°–74° angle (Figure 7B, lower graphs). However, the long axis of *ena* mutant SGPs remained aligned with the AP axis of the gonad (Figure 7B, lower graphs). These results suggest that changes in both cell morphology and cell orientation contribute to gonad compaction in wild type and are dependent on Ena activity.

### The *ena* mutation alters intracellular localization of DE-cad in the SGPs

**Figure 8:**
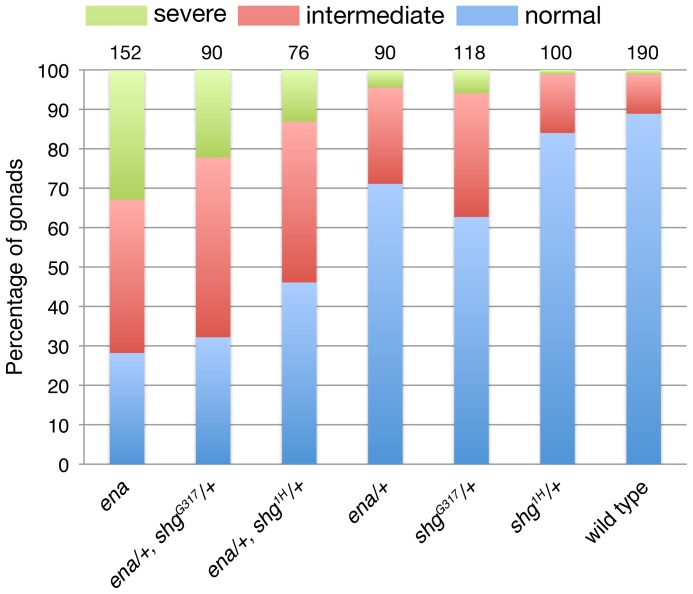
*ena* shows moderate genetic interaction with DE-cad in gonad compaction. To test for genetic interactions between *ena* and *DE-cad (shg)*, the gonad coalescence phenotype was examined in transheterozygotes of *ena* with *shg* mutant alleles. The coalescence phenotype at stages 15–16 was categorized into severe (green), intermediate (red), and normal classes (blue). *ena* and *shg* showed weak genetic interaction in gonad compaction. The number of gonads scored is indicated above the graph.

Ena has been shown to regulate the actin cytoskeleton at E-cadherin adhesive contacts [26], and *DE-cad/shotgun (shg)* mutants exhibit defects in gonad formation similar to those observed in *ena* mutants [13]. To determine if *DE-cad/shg* and *ena* genetically interact, we examined the phenotypes of embryos transheterozygous for *ena* and *DE-cad/shg*. We detected weak genetic interaction between *ena^C14-06^* and either *shg^G317^* or *shg^1H^*. In *ena^C14-06^/+, shg^G317^/+* transheterozygous embryos, 67.8% of gonads did not form correctly, compared to 28.8% *ena^C14-06^* heterozygotes and 37.3% for *shg^G317^*, suggesting that *ena* cooperates with *DE-cad/shg* in gonad compaction (Figure 8). We also analyzed other components of adherens junctions, such as β-catenin (*armadillo*) and Non-muscle Myosin II (*zipper*), in interaction with *ena*. Heterozygosity for either gene in combination with *ena* mutants increased in the penetrance and severity of the compaction phenotype compared to each single mutant (Figure S2). 51.9% of *arm^XK22^/+, ena^C14-06^/+* transheterozygous gonads and 59.8% of gonads in *ena^C14-06^/+, zip^1^/+* transheterozygous gonads failed to compact properly compared to 22.1% in *arm^XK22^* heterozygotes and 33.3% in *zip^1^* heterozygotes alone. The percentage of embryos with severe gonad coalescence phenotypes were significantly higher than those observed for the single mutants. The observed increase in gonad coalescence defects can, however, be explained by additive effects of mutating two parallel pathways both involved in gonad compaction rather than revealing a striking synergistic effect, which would suggest a more direct functional connection between Ena regulation and adherens junction components.

**Figure 9:**
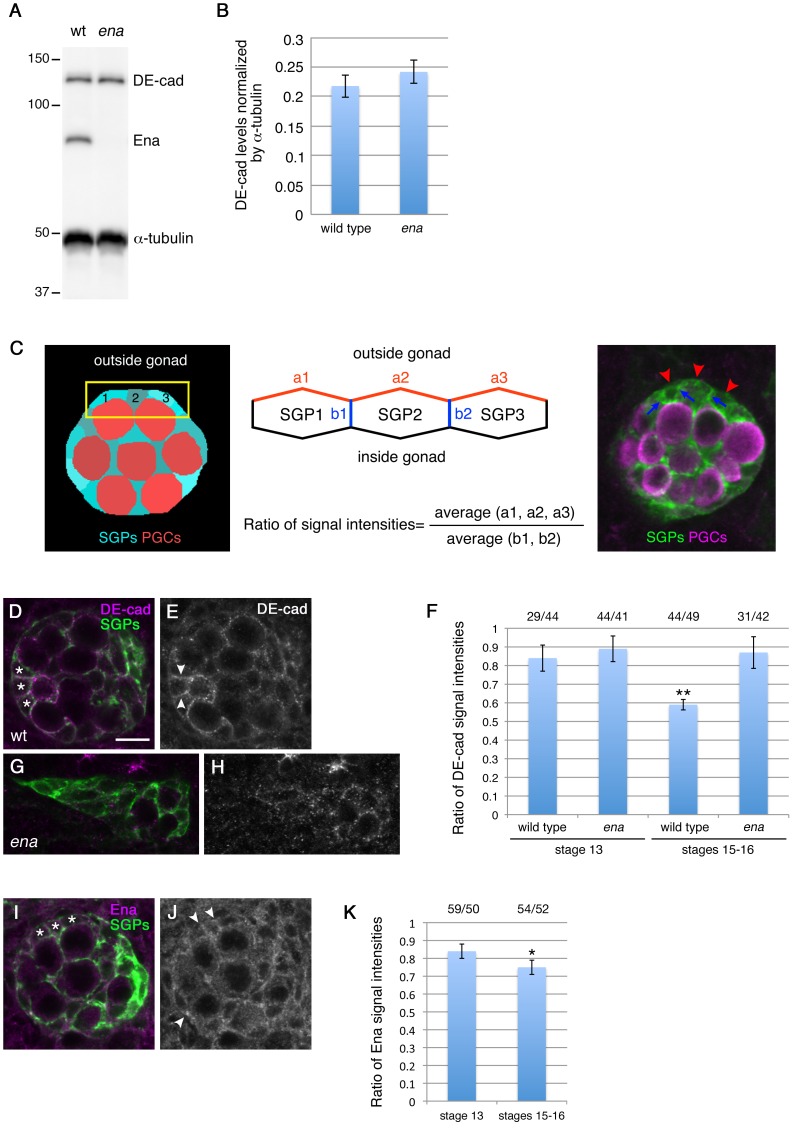
The *ena* mutation alters intracellular localization of DE-cad in SGPs. (A, B) The DE-cad protein in control and *ena* mutant embryos was detected by Western Blot (A) and the expression level of DE-cad normalized against α-tubulin expression was plotted in the graph (B). (C–H) Wild-type and *ena* mutant SGPs were stained with the DE-cad antibody, and the signal intensity on the outer membrane (red) or SGP-SGP boundary (blue) was measured (middle and left panels). The ratio of the signal intensity on the outer membrane compared to that on the SGP-SGP boundary was calculated at stage 13 and stages 15–16 (middle panel). In wild type, DE-cad becomes enriched at the SGP-SGP boundary as gonad compaction proceeds (**P<0.01) (D–F). This process was affected in *ena* mutants (F–H). (I–K) Wild-type SGPs were stained with the anti-Ena antibody (I, J), and the ratio of the signal intensity on the outer membrane compared to that on the SGP-SGP boundary was plotted at stage 13 and stages 15–16 (K). Ena localization shifts towards the SGP-SGP boundary at stages 15–16 (*P<0.05). The number of outer membranes versus SGP-SGP boundaries scored is indicated above the graph (F, K). Error bars in (B, F, and K) represent standard error.

Thus, to determine more directly whether Ena affects DE-cad during gonad compaction, we tested if *ena* mutations affect DE-cad protein expression and distribution. We found that DE-cad levels were unaffected in the mutant (Figure 9A and B). We then examined the intracellular localization of the DE-cad protein in wild-type and mutant SGPs. We stained embryos with a DE-cad antibody, and compared the signal intensity at the SGP membrane facing the outside of the gonad (the outer membrane, red in Figure 9C) and at the SGP-SGP boundary (the inner surface, blue in Figure 9C). In the wild type, DE-cad was localized relatively uniformly at the SGP membrane at stage 13 (Figure 9F). At stages 15–16, DE-cad was significantly enriched at the inner surface (P<0.01, Figure 9D–F). We also found that Ena localization gradually shifted from the outer membrane to the inner surface during gonad compaction (P<0.05, Figure 9I–K). In *ena* mutants, however, localization of DE-cad in the SGPs was not changed between stage 13 to 16 (Figure 9F–H). These data suggest a more direct interaction between Ena and DE-cadherin in gonad morphogenesis that was not revealed by the genetic interaction experiments.

### Mathematical simulation of gonad compaction

**Figure 10:**
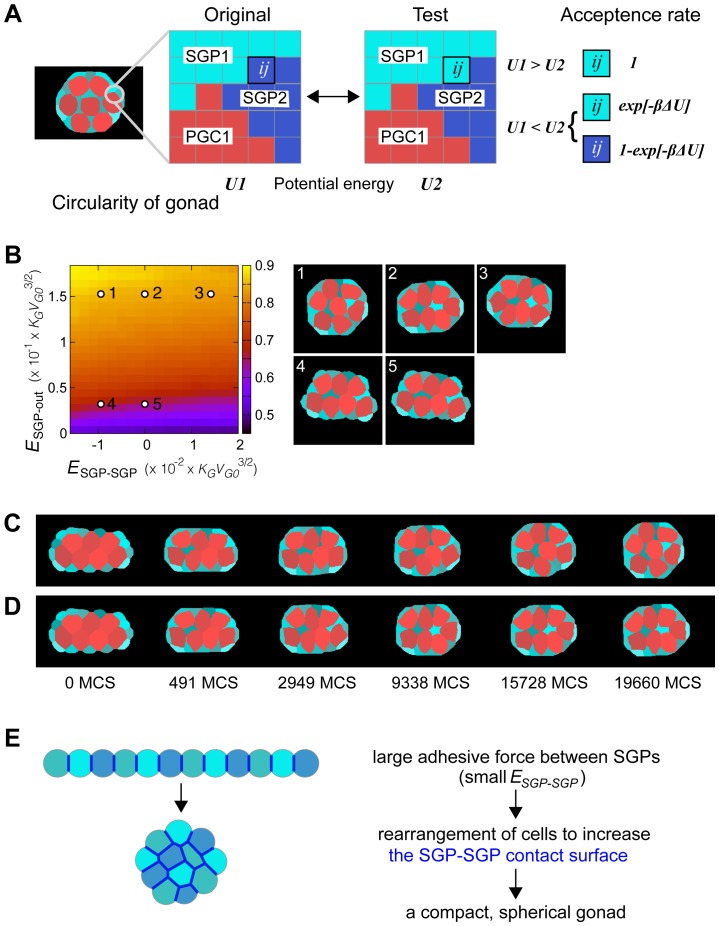
Mathematical simulation of gonad compaction. (A) A scheme of the Cellular Potts model (CPM). In the CPM, each cell is described over a region of multiple sites on a square lattice, and the shape of individual cells is determined by simulating the minimization process of a given potential energy for the system (see Materials and Methods for details). Briefly, in each Monte Carlo step (MCS), a flip of a randomly chosen pixel at the cell boundary (see the *ij*-th pixel) is adopted with the probability determined by the difference in potential energy between the two configurations. (B) Left: The average values of gonad compaction (circularity) between 12,000 and 20,000 MCS were calculated, and are shown in a color map against different values of *E_SGP-SGP_* and *E_SGP-out_*. The circularity increases for smaller contracting forces (larger adhesive forces) among SGPs, and for larger contracting force along the surfaces between SGPs and the outer space. Right: The cell configurations obtained from the CPM at 19,660 MCS. Values of *E_SGP-SGP_* and *E_SGP-out_* are indicated with the corresponding number in the left panel. Other parameter values are described in Methods. (C–D) Temporal changes in gonad morphology in the CPM. Selected frames from Movies S4 (C) and S5 (D) are shown. (C) and (D) correspond to 1 and 2 in the phase diagram in (B). (E) A mechanical view of gonad compaction. For clarity, only SGPs are shown. Blue lines indicate the SGP-SGP contact surface. Redistribution of DE-cad to the SGP-SGP surface generates a large adhesive force between the SGPs (i.e., small *E_SGP-SGP_*), which increases the SGP-SGP contact surface. The adhesive force, therefore, functions as a glue to move the SGPs inward (left).

It has been shown that the acto-myosin network generates the cell-surface contraction force and that cell adhesion, which favors a longer cell-cell surface, opposes the effect of the acto-myosin network [36], [37]. Our results suggest that the relocation of DE-cad changes the force balance in the SGPs, leading to the compaction of the gonad. To test this hypothesis, we developed a mathematical simulation that models gonad coalescence according to the parameters observed. A coefficient of linear interfacial energy (*E_j_*) represents linear tension. The acto-myosin network contracts the membrane and increases *E_j_*, while cell adhesion extends the contact surface and decreases *E_j_*. As described above, in wild-type embryos, DE-cad was up-regulated at the inner surface of the SGPs (Figure 9D–F). These observations suggest that *E_SGP-SGP_* decreases as wild-type gonads compact, while remaining unchanged in *ena* mutant gonads. Based on these assumptions, we conducted a mathematical simulation of a mechanical model to test if the observed changes in DE-cad localization can explain the compaction process (Figure 10A and Materials and Method). As shown in Figure 10B-D, our numerical simulation recapitulated the gonad compaction *in vivo* (see also Movies S4 and S5). By changing the *E_SGP-SGP_* values, we found that gonad circularity increased for smaller contraction forces (larger adhesive forces) between SGPs (Figure 10B). These results suggest that DE-cad may generate the forces required for the compaction of an elongated structure into a spherical gonad through the control of adhesion between SGPs, and the failure of gonad compaction in *ena* mutants can be explained by alterations in the force balance resulting from DE-cad mislocalization in the SGPs.

## Discussion

SGPs and PGCs display dramatic changes as they progress from cell aggregates to the well-compacted embryonic gonads. In the mature gonad, PGCs are embedded in a mesh network of SGP cytoplasmic extensions, and the interaction between these cells is enhanced by SGP-driven gonad compaction. The compaction process is important not only in maintaining the structural integrity of the gonad but also in ensuring proper differentiation of germline stem cells, which requires the intimate association of PGCs and the niche derived from anterior SGPs (reviewed in [38]). In this study, we found that Ena, an actin regulator, is required for gonad compaction. Ena acts in the soma, and loss of *ena* alters SGP cell shape and orientation, and intracellular distribution of DE-cad. Our mathematical simulation suggests that Ena has an important role in regulating the cadherin-dependent cell adhesive forces necessary for proper organ morphogenesis.

### Regulation of SGP cell shape and orientation during gonad compaction

Our detailed analysis of SGP morphology during gonad formation revealed that gonad compaction relies on coordinated changes in SGP cell shape and orientation. Anterior SGPs turn less than those located in the middle or posterior pole, which may contribute to the overall anterior movement of the gonad primordium from its original position at PS10-12 to its final location at PS10-11 [8]. Consistent with their failure to form a compact gonad, the angle of *ena* mutant SGPs is smaller with respect to the gonad AP axis (Figure 7B).

We noticed that the *ena* mutation affects SGP cell shape and axis orientation differently in different regions of the gonad. Defects in cell shape changes were more severe at the anterior than in the posterior (Figure 7A), while cell orientation was disturbed more severely in the middle and posterior SGPs (Figure 7B). Since *ena* is detected uniformly in all SGPs, these phenotypic differences are likely due to internal differences in the SGPs. In the early stages of development, SGPs are specified as three clusters in PS10, 11, and 12 in the mesoderm. The homeobox gene, *abd-A*, is expressed in PS10 through 12, while *abdominal-B (abd-B)* is only expressed in PS11 and 12 at the coalescence stage [39]. Although the ultimate fate of individual SGPs from each parasegment remains unknown, differential expression of *abd-A* and *abd-B* could account for the different cellular behaviors.

### Regulation of SGP polarity

It is well established that Ena family proteins promote actin filament elongation [29]. Therefore, it is likely that Ena is involved in DE-cadherin dynamics through regulation of the local actin morphology in SGPs. Ena translocation to the inner surface of SGPs (Figure 9I–K) might accelerate actin filament bundling at the inner surface. DE-cad is known to associate with bundled actin filaments and this may contribute to its accumulation at the inner surface of SGPs. Mechanical force could also be involved in DE-cad localization. *In vitro* analysis has demonstrated the force-dependent recruitment of the actin-binding protein, Vinculin, by α-Catenin. Adhesion by E-cad to the adjacent cell stretches the membrane, with the apparent transmission of the stretching force to α-Catenin resulting in the recruitment of Vinculin and actin filaments [40]. This mechanism could also act at the contact surface between SGPs, thus stabilizing DE-cad.

### Forces underlying gonad compaction

Recent studies have shown that cellular pattern formation during morphogenesis is coordinated via the localization and/or activity of force-generating molecular machinery, such as cell adhesion molecules and Myosin [36], [37]. Thus, the altered DE-cad distribution in *ena* mutants prompted us to examine whether *ena* controls the force balance in gonadal cells by regulating DE-cad. Since *ena* expression and function are necessary in the soma, and PGCs are dispensable for gonad compaction, it is reasonable to focus on SGPs to determine the parameters controlling these forces (*E_SGP-out_* and *E_SGP-SGP_*) by numerical simulation. Our *in silico* analysis showed that gonad compaction is promoted by increased adhesive force on the inner surface (smaller *E_SGP-SGP_*). This is consistent with the DE-cad relocation observed *in vivo*. Larger adhesive forces between SGPs increase SGP-SGP adhesion surfaces leading to SGP rearrangement and incorporation to form a spherical gonad (Figure 10E). We also showed that a larger contraction force along the outer surface (between the SGP and surrounding non-gonadal environment) (larger *E_SGP-out_*) resulted in increased gonad compaction; however, no significant Myosin II accumulation at the outer membrane of SGPs was detected (data not shown). One possibility is that Myosin activity, instead of localization, is increased at the outer membrane. Alternatively, Myosin II could act in the SGP cytoplasmic extensions to contract them, thereby generating the inward force required for gonad compaction. However, no overt changes were observed in the cellular protrusions in *ena* mutants (Figure 6E–H), making this alternative less likely.

Interestingly, Weyers *et al.* (2011) found that *robo* genes are required for gonad compaction and Robo2 was localized at contact sites between SGPs [16]. The Robo receptor family is reported to be involved in homophilic and heterophilic adhesion and repulsion [41], [42], [43]. Indeed, Ena has been shown to bind to Robo [44], suggesting that the observed effects of Robo on gonad morphogenesis could be mediated by Ena. Ena's function appears, however, primarily associated with the morphogenetic movements and cell shape changes observed during gonad formation, as *ena* mutants, in contrast to *robo* mutants, do not affect PGC ensheathment. Future quantitative measurement of force dynamics and force-generating molecular machinery, coupled with live observation in specific genetic backgrounds, will further clarify the mechanics of gonad compaction.

## Materials and Methods

### Mutagenesis

The C14-06 mutation was identified in a maternal screen on the right arm of the second chromosome [28]. In this screen, we crossed germline clone females to their siblings, resulting in the isolation of both maternal and zygotic mutants. The gonad morphogenesis phenotype of C14-06 was purely zygotic without any maternal effects. By deficiency mapping, we found that Df(2R)P34 and Df(2R)Exel6069 uncovered the C14-06 mutation. Since these deficiencies have an overlap between 56B5 and 56C1, we conducted a complementation test using mutations located in this region. We found that the C14-06 mutant did not complement the gonad morphogenesis defects and lethality of *ena^23^, ena^210^, ena^GC1^*, or *ena^GC5^*, indicating that *ena^C14-06^* is an allele of *ena*. The order of phenotype severity was as follows: *ena^C14-06^/Df, ena^C14-06^/ena^C14-06^>ena^GC1^/ena^C14-06^, ena^GC5^/ena^C14-06^>ena^23^/ena^C14-06^, ena^210^/ena^C14-06^*, which is consistent with the previously reported strength of *ena* alleles [45], [46]. Sequencing of the mutant allele identified a C-to-T change resulting in the alteration of Q388 to a stop codon.

### Fly Stocks

*ena^23^* has two mutations; a missense mutation in the proline rich-domain and a nonsense mutation in the EVH2 domain of the Ena protein (Ahern-Djamali *et al.*, 1998), whereas *ena^210^* has a missense mutation in the EVH1 domain [46]. *ena^GC1^* and *ena^GC5^* are strong alleles of *ena* generated by gamma-ray irradiation [45]. *shg^1H^* is a strong loss-of-function allele of *DE-cad* [47], [48]. It has been reported that *shg^G317^* shows a stronger phenotype than *shg* null alleles, suggesting that *shg^G317^* could be a dominant-negative allele of *shg/DE-cad* [47], [13]. *arm^XK22^* and *arm^XM19^* are null alleles of the *arm*/β-*catenin* gene with nonsense mutations [49]. *zip^1^* and *zip^2^* are null alleles of the *zip*/*Myosin II* gene [50]. Embryos from *osk^CE4^/osk^301^* females develop a normal abdomen but lack PGCs at 18°C [31]. For the rescue experiments, *nos*-GAL4VP16 [51] and 24B-Gal4 [52] were used in combination with UAS-*ena* [45], these constructs did not show a gonad compaction phenotype when tested separately (Figure 4B; data not shown).

### Western blotting

Extracts from stage 13 to 16 embryos were run on SDS-PAGE. The blotted membrane was incubated with mouse anti-Ena monoclonal antibody (5G2, 1∶500; C. Goodman, DSHB) generated against the N terminus of Ena [44], mouse anti-GFP monoclonal antibody (1∶1,000; Clontech), and rabbit anti-β-tubulin antibody (1∶10,000; Jackson ImmunoResearch). The signals were detected with the ECL system (GE Healthcare). For the quantification of DE-cad, extracts from stage 15–16 embryos were used. The primary antibodies used were rat anti-DCAD2 (1∶50; DSHB), mouse anti-Ena monoclonal antibody (1∶500), and anti-α-tubulin antibody (1∶12,000; Sigma). DE-cad signals were normalized against the signals of α-tubulin.

### Immunostaining and *in situ* hybridization

Antibody staining was conducted as described previously [11], using the following primary antibodies: mouse anti-Ena (5G2, 1∶200; C. Goodman, DSHB), rabbit anti-Vas (1∶20,000; Lehmann, R.), chick anti-Vas (1∶300; Lehmann, R.), rabbit anti-Zfh-1 (1∶4,000; Lehmann, R.), rabbit anti-Serpent (1∶500; D. Hoshizaki), rabbit anti-GFP (1∶1,000; Clontech), rat anti-DE-cad (DCAD2, 1∶50; T. Uemura, DSHB). RNA probes of the 412 retrotransposon were used to detect SGPs *in situ*, as described in [53].

### Construction of the transgene and transgenic lines

The third intron of the *D-six4* gene was cloned into a modified pGreen Pelican vector without GFP (pD-Six4III Colorless Pelican) [2]. The *egfp* gene linked to the actin-binding domain of *moesin* were cloned into the *Kpn* I/*Not* I sites of pD-Six4III Colorless Pelican to generate the P*_D-six4_-egfp::moesin* transgene. The P*_D-six4_-egfp::moesin* transgene was integrated into the fly genome by P-element-mediated transformation.

### Live imaging of the SGPs

SGPs were labeled with the P*_D-six4_-egfp::moesin* transgene, and live imaging was conducted as described previously [4]. For Figure 5A–I, images were taken with a 40× objective lens (Nikon, Plan Fluor, water, NA 0.75) on a BioRad Radiance Multiphoton system with a Nikon Eclipse E600FN microscope and a Tsunami laser (Spectra physics). For Figure 5J–O, we utilized a 40× objective lens (Olympus, UPlan FL, oil, NA 1.42) on a Prairie Technologies Ultima Multiphoton system with an Olympus BX51 W1 microscope and a Coherent Chameleon Ultra 1 80 MHz Ti: Sapphire laser. In Movies S1 and S2, the wild-type embryos were filmed every 2 minutes, while the *ena* mutant embryos in Movie S3 were filmed every 3 minutes. The Volocity 2.5.1 software (Improvision) was used to reconstruct images into three-dimensional movies.

### Analysis of cell shape and orientation

Images were taken by LSM700 confocal microscope (Zeiss). The maximum projection function of the IMARIS software (Bitplane) was used to visualize the cytoplasmic protrusions from the SGPs across the entire gonad. To measure SGP circularity, we used a single plane, which gives the largest nuclear diameter (This allowed us to examine approximately the same position in all cells). We measured SGP area and circumference, and calculated circularity as 4π*(area/circumference^2^) on the Image J software (NIH). A circularity value of 1.0 indicates a perfect circle, and an elongated polygon approaches the value 0. For cell orientation, we measured the angle between the longest cell axis and the AP axis of the gonad in 2D.

### Measurement of DE-cadherin levels in the SGPs

Embryos with the P*_D-six4_-egfp::moesin* transgene were stained with anti-DE-cad and anti-GFP antibodies and were scanned with LSM700 confocal microscope (Zeiss). Signal intensities of DE-cad on the SGP membrane were measured by the ImageJ software (NIH). The ratio of DE-cad signals in the outer surface to those in the SGP-SGP boundary was calculated on the same section.

### Mathematical simulation of gonad compaction

A 2D Cellular Potts model (CPM) was employed to simulate gonad morphogenesis, where each cell is described over a region of multiple sites on a square lattice, and the cell configuration is determined by simulating a relaxation (decrease) of a given potential energy (see below) [54]. The perigonadal environment is treated as a medium. The potential energy of the system depends on cell morphology and is assigned to each cell and interface, as *U = Σ_cells_U_vol_*(*V_i_*)*+Σ_interfaces_U_lin_*(*l_j_*)*+Σ_cells_U*_quad_(*L*_i_), where *V_i_* and *L_i_* represent the volume and peripheral length of the *i*-th cell, respectively, and *l_j_* is the cell-cell interface or outer membrane length. *U*_vol_(*V*_i_) = (*K_i_*/2)(*V*_i_−*V*_i0_)^2^ represents the elastic energy of a cell, where *K_i_* and *V_i0_* are the elastic coefficient and favored volume of the *i*-th cell, respectively. *U_lin_*(*l_j_*) = *E_j_l_j_* is the linear part of interfacial energy, which is determined by the balance between cell-cell adhesion and cortical tension. *U_quad_*(*L_i_*) = (*C*_i_/2)*L*_i_^2^ is the quadratic part of the energy, representing the contribution of cortical cell tension as a spring [55], [56].

The relaxation of the potential energy is performed by the Metropolis algorithm (Figure 10A) [37], [54], [56], which is a commonly used Monte Carlo method. The Metropolis algorithm is the iteration of the following procedures. First, as a test configuration, a randomly chosen lattice point at the cell boundary is flipped to an adjacent cell (see the *ij*-th pixel in Figure 10A). Then, values of the potential energy are compared between the original and test cell configurations by calculating *ΔU = U*_test_ *−U*_orig_. The test configuration is adopted with probability *p* = 1 for *ΔU*≤0 and *p* = exp [*−βΔU*] for *ΔU*>0. Here, ‘inverse temperature’ *β* controls the randomness of the cell morphology. One Monte Carlo step (MCS) is composed of iterations of these procedures lattice-size times. Repeating MCS calculates temporal changes in cell shapes (Figure 10C and D, Movies S4 and S5).

We simulated a system composed of 7 PGCs and 14 SGPs. Parameters were set as *K_G_* = 80.0, *V_G0_* = 1600, *K_S_* = 200.0, *V_S0_* = 400, *C_G_* = 6.8×10^−3^×(*K_G_V_G0_*), *C_S_* = 7.4×10^−3^×(*K_G_V_G0_*), *E_PGC-PGC_* = 4.0×10^−2^×(*K_G_V_G0_^3/2^*), *E_PGC-SGP_* = −2.0×10^−2^×(*K_G_V_G0_^3/2^*), *E_PGC-out_* = 2.2×10^−1^×(*K_G_V_G0_^3/2^*), and *β* = 1.2×10^−5^. Note that DE-cad might simply extend the contact surface between cells (a smaller *E_SGP-SGP_*), or recruit Myosin through actin filaments leading to membrane contraction (a larger *E_SGP-SGP_*). Our observations support the former possibility. Myosin was not elevated in the SGP-SGP contact surface (data not shown), and the relative values for tension, measured by responses to cutting a contact surface (laser ablation was done as described in [57], were smaller in the SGP-SGP surface than in the outer surface (data not shown). Gonad circularity was evaluated by the 10 trials average measured between 12,000 and 20,000 (MCS) in each simulation.

## Supporting Information

Figure S1 **Expression pattern of the P** ***_six4_-egfp::moesin*** **transgene.** The EGFP::Moesin fusion protein was detected by the anti-GFP antibody (green). PGCs were labeled by the anti-Vas antibody (magenta). The fusion protein is expressed broadly in the mesoderm at stage 11 (A). The expression is gradually restricted in the SGPs during stage 13 to 16 (B, C).
(TIF)

Figure S2 **Gonad compaction phenotype in transheterozygotes of** ***ena*** **with** ***arm/β-cat*** **or** ***zip/Myosin II*** **.** Gonad coalescence phenotype was examined in transheterozygotes of *ena* with *arm/β-cat* or *zip/Myosin II* mutant alleles. The coalescence phenotype at stages 15–16 was categorized into severe (green), intermediate (red), and normal classes (blue).
(TIF)

Movie S1 **Live imaging of the wild-type gonad at early stage 13.** SGPs were labeled with the P*_six4_-egfp::moesin* transgene. Images were taken by time-lapse two-photon microscopy using a BioRad Radiance Multiphoton system with a Nikon microscope (Eclipse E600FN). Frames were taken every 2 minutes. Selected still images of this movie are shown in Figure 5A–C.
(MOV)

Movie S2 **Live imaging of the wild-type gonad at middle stage 13 to stage 14.** SGPs were labeled with the P*_six4_-egfp::moesin* transgene. Images were taken by time-lapse two-photon microscopy using a BioRad Radiance Multiphoton system with a Nikon microscope (Eclipse E600FN). Frames were taken every 2 minutes. Selected still images of this movie are shown in Figure 5D–I.
(MOV)

Movie S3 **Live imaging of the** ***ena*** **mutant gonad at middle stage 13 to stage 14.** SGPs were labeled with the P*_six4_-egfp::moesin* transgene. Images were taken by time-lapse two-photon microscopy using a Prairie Technologies Ultima Multiphoton system with an Olympus microscope (BX51W1). Frames were taken every 3 minutes. Selected still images of this movie are shown in Figure 5J–O.
(MOV)

Movie S4 **Patterns obtained by the CPM model.** Parameter values are *E_SGP-SGP_* = −0.01 and *E_SGP-out_* = 0.16. Other parameter values are described in Method. Six frames of this movie are shown in Figure 9C.
(MOV)

Movie S5 **Patterns obtained by the CPM model.** Parameter values are *E_SGP-SGP_* = 0 and *E_SGP-out_* = 0.16. Other parameter values are described in Materials and Methods. Six frames of this movie are shown in Figure 9D.
(MOV)
