# Supplementary figures and images for: The Drosophila Actin Regulator ENABLED Regulates Cell Shape and Orientation during Gonad Morphogenesis
Source: PLoS One. 2012 Dec 26;7(12):e52649. doi: 10.1371/journal.pone.0052649 (PMC3530444; doi:10.1371/journal.pone.0052649)

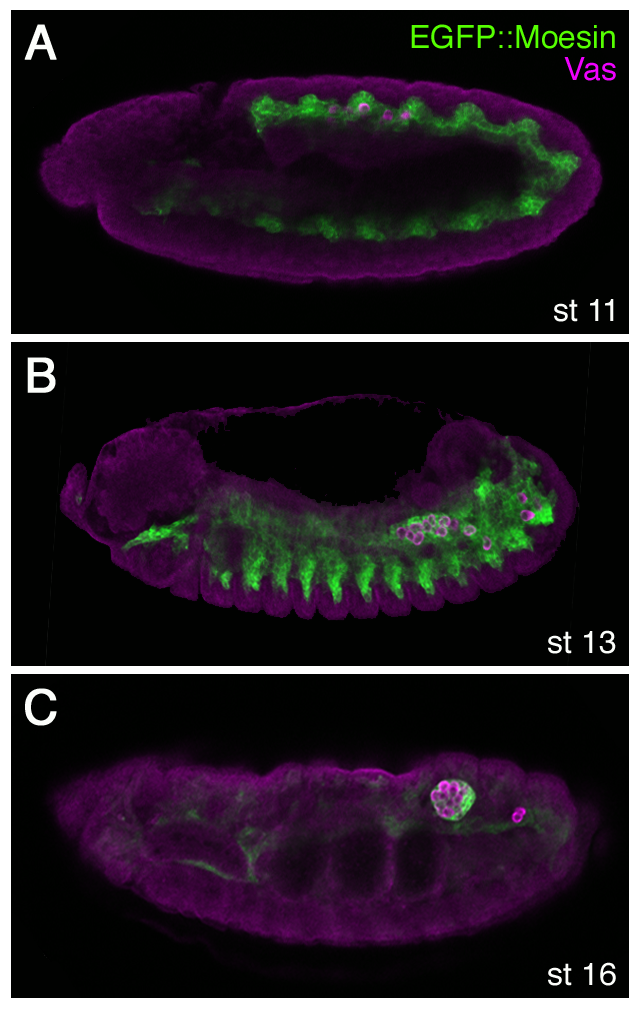

Supplement: Figure S1 — Expression pattern of the P six4-egfp::moesin transgene. The EGFP::Moesin fusion protein was detected by the anti-GFP antibody (green). PGCs were labeled by the anti-Vas antibody (magenta). The fusion protein is expressed broadly in the mesoderm at stage 11 (A). The expression is gradually restricted in the SGPs during stage 13 to 16 (B, C). (TIF) [file pone.0052649.s001.tif]

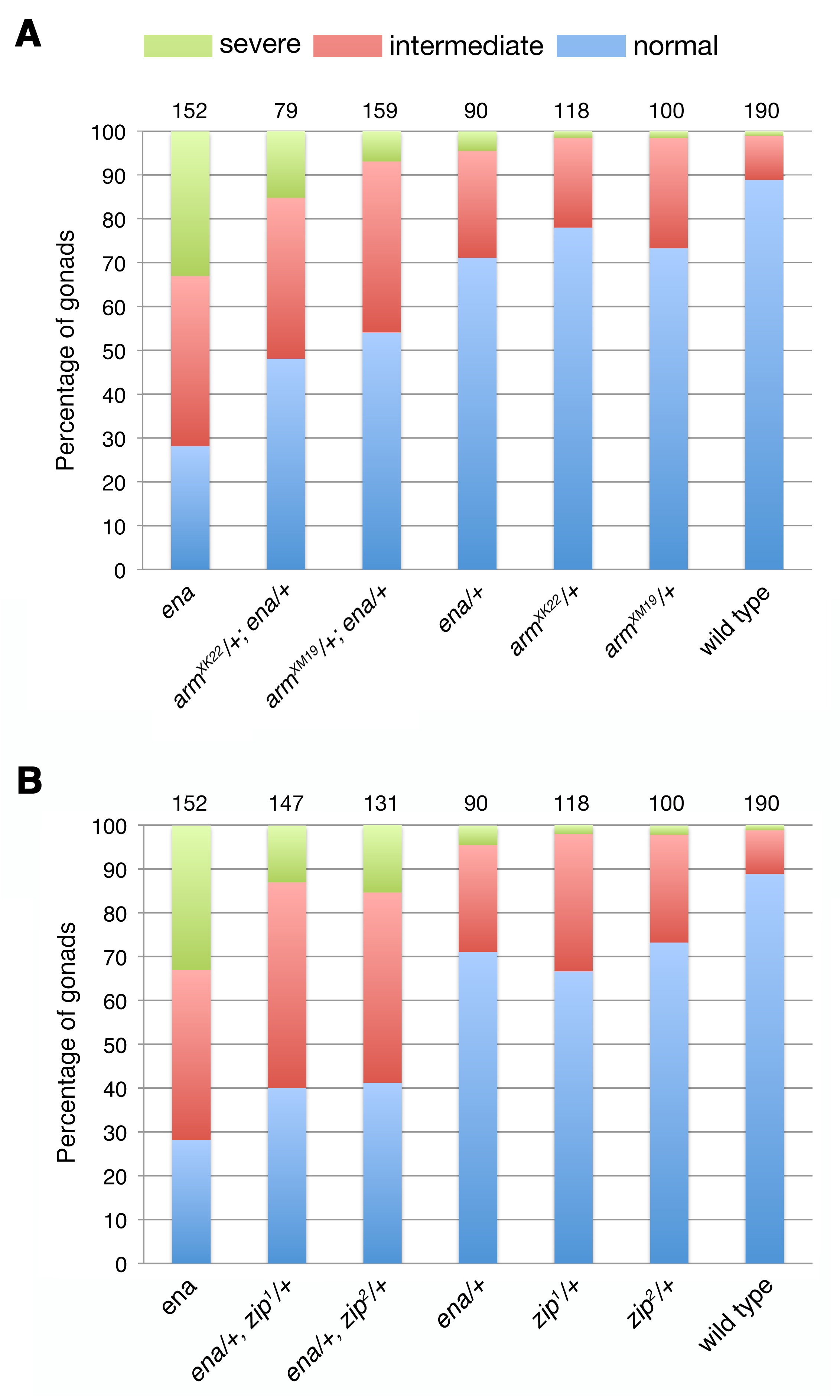

Supplement: Figure S2 — Gonad compaction phenotype in transheterozygotes of ena with arm/β-cat or zip/Myosin II . Gonad coalescence phenotype was examined in transheterozygotes of ena with arm/β-cat or zip/Myosin II mutant alleles. The coalescence phenotype at stages 15–16 was categorized into severe (green), intermediate (red), and normal classes (blue). (TIF) [file pone.0052649.s002.tif]
